# Synthetic Small Molecule Modulators of Hsp70 and Hsp40 Chaperones as Promising Anticancer Agents

**DOI:** 10.3390/ijms24044083

**Published:** 2023-02-17

**Authors:** Bianca Nitzsche, Michael Höpfner, Bernhard Biersack

**Affiliations:** 1 Institute for Physiology, Charité—Universitätsmedizin Berlin, Corporate Member of Freie Universität Berlin and Humboldt Universität zu Berlin, Charitéplatz 1, 10117 Berlin, Germany; 2 Organische Chemie 1, Universität Bayreuth, Universitätsstrasse 30, 95440 Bayreuth, Germany

**Keywords:** anticancer agents, heat shock proteins, Hsp70, Hsp40

## Abstract

A class of chaperones dubbed heat shock protein 70 (Hsp70) possesses high relevance in cancer diseases due to its cooperative activity with the well-established anticancer target Hsp90. However, Hsp70 is closely connected with a smaller heat shock protein, Hsp40, forming a formidable Hsp70-Hsp40 axis in various cancers, which serves as a suitable target for anticancer drug design. This review summarizes the current state and the recent developments in the field of (semi-)synthetic small molecule inhibitors directed against Hsp70 and Hsp40. The medicinal chemistry and anticancer potential of pertinent inhibitors are discussed. Since Hsp90 inhibitors have entered clinical trials but have exhibited severe adverse effects and drug resistance formation, potent Hsp70 and Hsp40 inhibitors may play a significant role in overcoming the drawbacks of Hsp90 inhibitors and other approved anticancer drugs.

## 1. Introduction

Stress factors such as heat lead to defensive and protective cellular responses, enabling the cell to cope with the stress [1]. The emergence of chromosomal puffs as a defined heat shock response in *Drosophila* flies was observed for the first time in 1962 by Ferruccio Ritossa [2]. In 1974, the increased expression of certain heat shock proteins in response to heat and other stress factors was discovered [3]. These heat shock proteins (Hsps) are classified by their molecular weights in kDa (e.g., Hsp90, Hsp70, etc.) and function as chaperones in order to protect important proteins from degradation, to control the quality of protein folding, and to deliver misfolded or damaged proteins to the proteasome for disposal (protein triage), thus vouchsafing cell viability under these conditions [4]. This multi-chaperone system (“epichaperome”) plays an important role in various cancer diseases [5]. Canonical functions of chaperones are linked with the ubiquitin-proteasome system (UPS) and chaperone-mediated autophagy (CMA), while non-canonical functions affect the immune system, including inflammatory and autoimmune mechanisms [6]. Thus, targeting heat shock proteins is a promising strategy to combat cancer.

The Hsp family is subdivided into Hsp110 (HSPH), Hsp90 (HSPC), Hsp70 (HSPA), Hsp40 (DNAJ), small Hsps (HSPB), and the chaperonin family proteins Hsp60/Hsp10 (HSPD/E) and TRiC (CCT) [7]. Hsp90 has already become a valuable chaperone drug target. Prominent examples of anticancer active Hsp90 inhibitors are the natural products (geldanamycin and radicicol) as well as their (semi-)synthetic derivatives, 17-AAG/tanespimycin and ganetespib, the latter have reached clinical trials [8]. Hsp90 regulates the activity and stability of crucial transcription factors such as the tumor suppressor p53 and the androgen receptor (AR), while Hsp90 activity itself is regulated by posttranslational modifications (e.g., lysine acetylation under control by HDAC6) and by other heat shock proteins such as Hsp70 [9]. The protein folding by Hsp90 and Hsp70 is ATP-dependent, while Hsp70 has a crucial function in the protection of cells against various stress factors, including enhanced cell survival. In addition, Hsp70 forms complexes with Hsp90 with the help of HOP (Hsp70–Hsp90 organizing protein) in order to exert its housekeeping activities [10,11,12]. There is growing evidence that Hsp70 inhibitors have the potential to overcome Hsp90 inhibitor resistance [13]. The organelle-specific members of the Hsp70 protein family, such as mitochondrial mortalin (mtHsp70, Grp75) and endoplasmic reticulum/ER-based Grp78, were also identified as possible anticancer drug targets due to their crucial roles in cell proliferation and survival in various cancers [14,15]. In addition, extracellular Hsps attracted increased attention as cancer targets and biomarkers [16].

Client polypeptides are transferred to Hsp70 by the smaller co-chaperone, Hsp40. Hsp40 prevents the aggregation of unfolded polypeptides, but there are also folded proteins among the clients of Hsp40. There are numerous Hsp40 isoforms, also dubbed J-domain proteins (DNAJs), that are structurally different from Hsp70 and Hsp90 proteins [17]. Nevertheless, DNAJ/Hsp40 and Hsp70 proteins form a tight Hsp70/Hsp40 complex in order to fulfill their protein-folding functions [17,18]. Hsp40 binds to the nucleotide-binding domain (NBD) of Hsp70 and accelerates the ATPase activity of Hsp70 enormously [18]. Thus, small-molecule inhibitors of the Hsp70-Hsp40 axis have considerable potential as anticancer drug candidates.

In this review, the current state and recent developments in the field of synthetic Hsp70 and Hsp40 inhibitors are discussed.

## 2. Hsp70 Inhibitors

The mechanisms of action of proteins of the Hsp70 family and their roles in various cancer diseases were thoroughly reviewed recently [10,11,12]. In line with the mounting knowledge of these proteins, Hsp70 inhibitors are a continuously growing class of compounds, which can be subdivided into Hsp70 and Hsc70 inhibitors on the one hand and inhibitors targeting organelle-specific Hsp70 proteins such as Grp78 and mortalin on the other hand.

### 2.1. Hsp70i and Hsc70 Inhibitors

Inducible Hsp70 (also abbreviated as Hsp70i) and consecutive Hsc70 inhibitors can be classified according to their binding mode into N-terminal nucleotide binding domain/NBD-targeting inhibitors, C-terminal substrate binding domain/SBD-targeting inhibitors, and allosteric inhibitors (Table 1).

**Figure 1:**
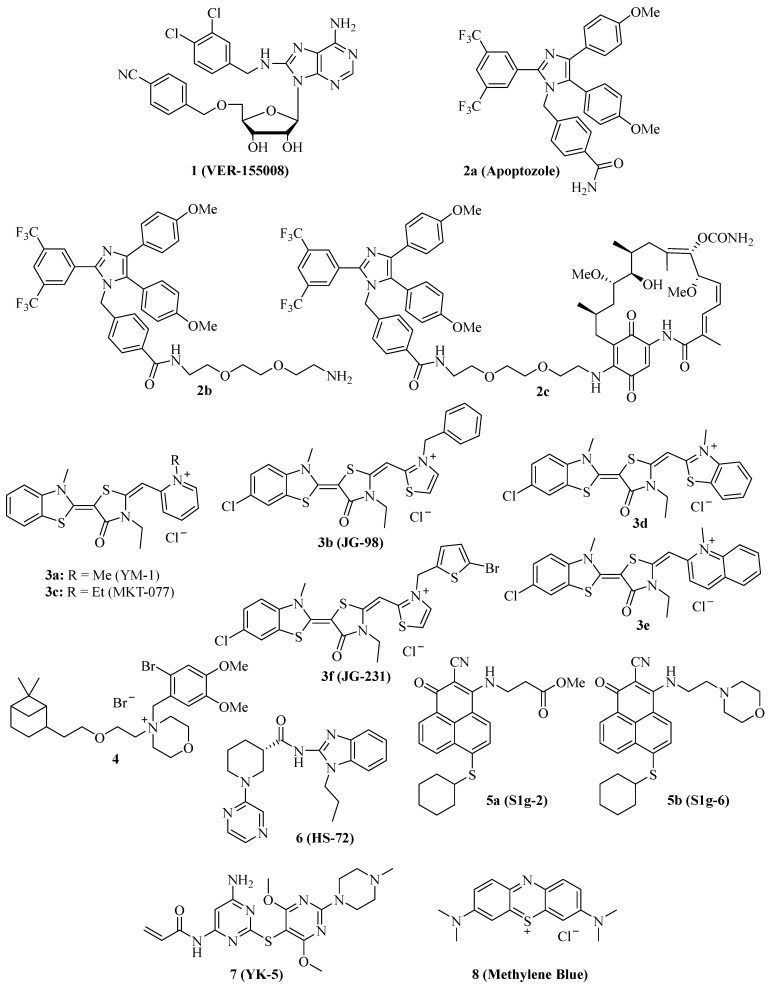
Structures of NBD-binding and interfering compounds **1**–**8**.

In 2009, the adenosine-derivative **1** (Figure 1, VER-155008) was identified as a selective NBD-targeting Hsc70/Hsp70 inhibitor (IC_50_ = 0.5 µM), which showed antiproliferative effects on HCT-116 colon carcinoma cells (GI_50_ = 5.0 µM), reduced Raf-1 and Her2 protein levels, and enhanced the apoptosis induction by the Hsp90 inhibitors 17-AAG and VER-82160 in HCT-116 cells [19,20]. Compound **1** also revealed promising effects on non-small cell lung cancer (NSCLC), such as inhibition of NSCLC proliferation and cell cycle arrest (increased G0/G1 cell percentage) [21]. In addition, compound **1** inhibited pleural mesothelioma cell proliferation and colony formation, which are associated with G1 cell cycle arrest, suppressed phospho-Akt, and induction of macroautophagy [22]. In LNCaP95 prostate cancer cells, the Hsp70 inhibitor **1** induced apoptosis and suppressed the expression of the full-length androgen receptor (AR-FL) and of the androgen receptor splice variant 7 (AR-V7), which are associated with castration-resistant prostate cancer (CRPC) [23]. These AR-suppressing effects were correlated with an inhibition of YB-1 phosphorylation by compound **1**, followed by reduced nuclear translocation of YB-1. Another study using the AR-positive LNCaP and the AR-negative PC-3 prostate cancer cell lines showed that compound **1** was antiproliferative and pro-apoptotic in both cell lines, albeit the pro-apoptotic effects were higher in the AR-positive cells [24]. Compound **1** downregulated AR expression and induced G1 cell cycle arrest in the LNCaP cells. Moreover, a distinct suppression was observed for Hsp27 in PC-3 cells and HOP and Hsp90β in both cell lines treated with compound **1**. In MCF-7 breast cancer cells, compound **1** induced apoptosis associated with mitochondrial damage, and the anticancer activity of compound **1** was reduced by heat shock [25]. Anaplastic thyroid carcinoma (APC) is the most lethal thyroid cancer with high drug resistance, but compound **1** was able to induce paraptosis in APC cells dependent on de novo protein synthesis [26]. In a panel of glioma cells (1321N1, GOS-3, and U87-MG), compound **1** showed higher antiproliferative activities (IC_50_ = 12–13 µM) than the approved drug temozolomide (IC_50_ = 135–180 µM) associated with downregulation of Akt kinase activity and the modulation of certain miRNAs, e.g., the upregulation of miR-215 and miR-194-5p [27]. Compound **1** was evaluated in muscle-invasive bladder cancer (MIBC) models and induced apoptosis along with inhibition of MIBC cell proliferation and migration [28]. The activity of compound **1** against MIBC was associated with the suppression of protein members of p53/Rb, PI3K, and SWI/SFW signaling. Especially strong degrading effects of compound **1** were observed on the demethylase KDMA6 and the histone acetyltransferase EP300, both members of the histone modification pathway.

Due to these promising anticancer effects of compound **1**, its combination with various other anticancer drugs was investigated. In combination with the Hsp90 inhibitor radicicol, compound **1** was able to enhance the APC cell killing activity of radicicol, accompanied by suppressed heat shock cognate 70/Hsc70, Akt, and survival [29]. The combination of compound **1** with the Hsp90 inhibitor STA9090 was evaluated in MIBC models and was more efficient than single compound therapy [28]. However, the combination of compound **1** with doxorubicin in canine osteosarcoma (OSA) cells showed no improvements. Already, compound **1** alone displayed strong apoptosis induction, inhibition of colony formation, and antiproliferative activities against OSA cells based on Hsp70 inhibition as well as Akt suppression and BAG1 degradation [30]. In contrast to that, compound **1** showed synergy effects in combination with the Hsp90 inhibitor 17-AAD and sensitized A549 NSCLC cells to radiation therapy [21]. The combination of compound **1** with manumycin A, an anticancer active antibiotic that upregulates Hsp70 in cancer cells, sensitized lung tumor cells to manumycin A treatment [31]. Micelles of compound **1** together with gold nanorods were successfully tested as mild-temperature photothermal therapy in colon cancer, leading to strong colon tumor growth in vivo at a temperature of 45 °C [32]. Compound **1** exhibited considerable activity against multiple myeloma (MM) cells (IC_50_ = 1.7 µM for OPM2, 3.0 µM for RPMI 8226, and 6.5 µM for MM.1S cells), and the combination of compound **1** with the proteasome inhibitor bortezomib displayed synergy effects in terms of apoptosis induction in MM cells, which was associated with suppression of anti-apoptotic Bcl-2, Bcl-xL, and Mcl-1 and upregulation of pro-apoptotic NOXA and Bim [33]. In addition, the ER stress marker CHOP (CCAAT-enhancer binding protein homologous protein) was induced by this combination treatment. The natural product shikonin was described as a proteasome inhibitor and necroptosis inducer in MM cells while it also upregulated Hsp70, and, thus, the combination with compound **1** enhanced shikonin-induced MM cell death [34]. Compound **1** also exhibited promising effects on acute myeloid leukemia (AML) cells alone (induction of apoptosis, inhibition of cell proliferation, and colony formation) and in combination with the Hsp90 inhibitor 17-DMAG (additive antiproliferative and pro-apoptotic activity) [35]. The release of AML cell growth factors and regulators such as TNF-α, VEGF, IL-3, IL-1β, and IL-1 receptor antagonist was strongly suppressed upon treatment with compound **1**. A new structurally related 6,8,9-trisubstituted purine derivative was recently disclosed that induced apoptosis and senescence in luminal A subtype MCF-7 breast carcinoma cells [36].

The substituted imidazole derivative **2a** (Figure 1, apoptozole) was discovered as a pro-apoptotic inhibitor of Hsc70 (K_D_ = 210 nM) and Hsp72 (K_D_ = 140 nM) in 2008, which showed high tumor cell growth inhibitory activities with GI_50_ values in the nanomolar concentration range (GI_50_ = 220 nM for SK-OV-3, 250 nM for HCT-15, and 130 nM for A549 cells) [37]. Compound **2a** was identified as an inhibitor of the Hsp70 ATPase by affinity chromatography upon conjugation of the amino-ethyloxy modified apoptozole derivative **2b** (Figure 1) with a resin, leading to a reduced interaction of Hsp70 with APAF-1, while no affinity to Hsp40, Hsp60, or Hsp90 was observed [38]. Time-dependent antiproliferative activity was checked, and amenable IC_50_ values (0.8 µM for A549, 0.8 µM for HeLa, 0.7 µM for MDA-MB-231, and 0.7 µM for HepG2 cells) were obtained after 72 h of incubation, while the combination with doxorubicin led to a sensitization of A549 and HeLa cells to doxorubicin treatment. At doses of 4 mg/kg/day (i.p.) given for two weeks, compound **2b** reduced the tumor growth of A549, RKO, and HeLa tumor xenografts by 61%, 65%, and 68%, respectively; in the latter case, the combination with doxorubicin led to more pronounced tumor growth inhibition (81% tumor growth reduction). The anti-leukemia properties of compound **2b** and its hybrid molecules, such as compound **2c** (Figure 1), with the Hsp90 inhibitor geldanamycin (**2b** and geldanamycin connected by ethylene glycol-based linker systems) were studied [39]. Compounds **2b**, hybrid **2c,** and other related hybrids induced apoptosis in a caspase-dependent way, however, the hybrids were more active against leukemia cells than their parent compounds **2b** and geldanamycin. Compound **2c** inhibited autophagy, but it also induced apoptosis in HeLa cancer cells based on its selective inhibition of the lysosomal Hsp70 and the degradation of lysosomal membranes associated with cathepsin release followed by caspase activation [40].

Several synthetic allosteric inhibitors of the Hsp70 ATPase domain were described. The rhodacyanine class of the Hsp70-inhibitory dye compounds was established by the discovery of compound **3a** (Figure 1, YM-1), in particular, by the blocking of the Hsp70 interaction with the nucleotide exchange factor (NEF) Bag3 by compound **3a** (IC_50_ = 4.8 µM) [41]. **3a** binds to an allosteric binding site, stabilizing ADP-bound Hsp70 with a weak Bag3 affinity. Consequently, compound **3a** suppressed FoxM1 and HIF1α pathways in MCF-7 and HeLa cells, which is unique for Hsp70-Bag3 inhibition since other Hsp70 inhibitors (e.g., the natural flavonoid myricetin) did not show such mechanistic effects. In MCF-7 breast carcinoma xenografts, compound **3a** (25 mg/kg every second day for 6 days, i.p.) inhibited tumor growth associated with induction of p21 and suppression of FoxM1 and survivin. The activity of compound **3a** was also investigated in glioma models, and it sensitized U251 and U343 glioma cells to treatment with the Bcl-2 inhibitors (-)-gossypol (AT-101) and ABT-737 [42]. Analogously, **3a** sensitized apoptosis-resistant and chemo-resistant breast cancer cells to drug treatment [43]. In doxorubicin-resistant BT-549^r^DOX cells, compound **3a** suppressed Mcl-1 and showed synergistic antiproliferative effects in combination with doxorubicin. In addition to compound **3a**, further close Hsp70-inhibitory analogs such as compound **3b** (Figure 1, JG-98) with thiazolium moieties were described, which were discovered during the search for optimized Hsp70 inhibitors derived from compound **3a** and the mortalin (mitochondrial Hsp70) inhibitor MKT-077 (Figure 1, compound **3c**, see below). Compound **3b** exhibited prolonged microsomal half-lives and at least 3-fold higher antiproliferative activities against MDA-MB-231 triple-negative breast cancer (TNBC; EC_50_ = 0.4 µM) and hormone-sensitive MCF-7 breast cancer cells (EC_50_ = 0.7 µM) than compounds **3a** (EC_50_ = 2.0 for MDA-MB-231 and 5.2 for MCF-7 cells) and **3c** (EC_50_ = 1.4 for MDA-MB-231 and 2.2 for MCF-7 cells) [44]. Compound **3b** induced apoptosis in MDA-MB-231 cells by caspase activation and enhanced p62 oligomerization as a hint at forming autophagosomes. Compound **3b** also inhibited the Hsp70-Bag3 interaction, which was accompanied by FoxM1 suppression and upregulation of p21 and p27 in treated MCF-7 cells [45]. Doses of 3 mg/kg (every second day for six days) of compound **3b** inhibited the growth of MCF-7 breast carcinoma xenografts in vivo. In addition to breast cancer cell lines MCF-7 and MDA-MB-231, further cancer cell lines sensitive to compound **3b** were identified (with EC_50_ < 1 µM), such as HeLa, A375, HT-29, SKOV-3, Jurkat, MM1.R, INA6, RPMI-8226, JJN-3, and U266. Mechanistically, compound **3b** exerted its antiproliferative activities against breast cancers both in Bag3-dependent (via ERK activation) and Bag3-independent ways (via suppressed Akt and *c*-Myc) [46]. Combinations with the proteasome inhibitors MG132 and bortezomib enhanced the antitumor activity of compound **3b** in breast cancer models both in vitro and in vivo (5 mg/kg of compound **3b** plus 1 mg/kg of bortezomib in MDA-MB-231 TNBC xenografts). Synergy effects in breast cancer cells were also observed in combination with α-amanitin (RNA-polymerase II inhibitor), LY294002 (Akt inhibitor), and sunitinib (RTK inhibitor). In addition, the infiltration of tumor bodies by tumor-associated macrophages (TAMs) was inhibited by compound **3b** based on the inactivation of tumor stromal cell Hsp70 proteins [47]. Similar to compound **1**, compound **3b** also sensitized lung cancer cells to treatment with manumycin A [31]. In a recent effort, new rhodacyanine analogs of compound **3b** with benzo-fused *N*-heterocycle moieties were described, with benzothiazolium compound **3d** and quinolinium compound **3e** (Figure 1) as the most promising compounds exhibiting high antiproliferative activity against TNBC cells (IC_50_ = 0.24 µM for **3d** and 0.37 µM for **3e** in MDA-MB-231 cells), accompanied by a considerable selectivity since non-malignant MCF-10A cells with low levels of Hsp70 were much less sensitive to treatment with **3d** [48]. Both compounds are very stable, with half-lives of more than 2 h in microsomes. Apoptosis upon caspase-activation was induced by compounds **3d** and **3e** in MDA-MB-468 breast cancer cells, while both compounds led to autophagy both in MDA-MB-231 and MDA-MB-468 cells. In addition to FoxM1, survivin, HuR, and Akt suppression, compounds **3d** and **3e** also degraded KRAS in MDA-MB-231 and MDA-MB-468 cells. Further **3b**/JG-98-derived benzothiazole rhodacyanines were described, culminating in the discovery of the bromothienyl analog **3f** (JG-231), which showed high activity against MCF-7 (IC_50_ = 0.12 µM) and MDA-MB-231 breast cancer cells (IC_50_ = 0.25 µM), disruption of Bag3 interaction, amenable microsomal stability (half-life of more than 60 min), degradation of Akt and HuR in MCF-7 xenografts, amenable in vivo pharmacokinetics parameters and in vivo tumor growth inhibition of MDA-MB-231 xenografts at doses of 4 mg/kg (i.p.) [49].

The cationic spasmolytic drug pinaverium bromide (Figure 1, **4**) was repurposed as an inhibitor of constitutively activated Hsc70. Compound **4** inhibited cell proliferation (IC_50_ of ca. 10 µM) of A2058 melanoma cells and induced apoptosis in these cells [50]. Its binding site was located at the NBD and linker domains of Hsc70.

S1g-2 (Figure 1, **5a**) was identified as an inhibitor of Hsp70-Bim interaction (IC_50_ = 0.4 µM) in CML cells by screening a Bcl-2 inhibitor library [51]. Its allosteric Hsp70-binding site is near the binding site of the rhodacyanines **3**. Compound **5a** selectively induces apoptosis in CML cells by suppressing oncoprotein clients such as Akt, Raf-1, eIF4E, and RPS16. Hsp70-Bim interaction protected BCR-ABL-independent TKI-resistant CML cells from apoptosis, and, thus, treatment with compound **5a** can overcome the resistance of a highly problematic CML type. The close analog compound **5b** (Figure 1, S1g-6), which has a morpholino side chain replacing the unstable ester side chain of **5a**, was recently described as a new sub-micromolar Hsp70-Bim interaction inhibitor [52]. Compound **5b** also induced apoptosis in cancer cells and suppressed Akt and Raf-1.

The benzimidazole derivative **6** (Figure 1, HS-72) selectively inhibits inducible Hsp70 (Hsp70i), which is in stark contrast to its low affinity for the closely related constitutively activated Hsc70 [53]. At doses of 25 and 50 µM, compound **6** inhibited the proliferation of BT474, MCF-7, and SkBr3 breast cancer cells, and the proteins Her2 and Akt were degraded by compound **6** in BT474 and MCF-7 breast cancer cells. In the Her2-overexpressing MMTV-*neu* spontaneous breast tumor mouse model, compound **6** (20 mg/kg biweekly for 21 days, i.p.) was well tolerated and inhibited breast tumor growth, leading to prolonged survival of treated mice.

The 2,5′-thiodipyrimidine **7** (Figure 1, YK-5) was identified as an irreversible inhibitor of a new allosteric site of the Hsp70 NBD domain and binds covalently to a cysteine residue of the binding site via its reactive acrylamide moiety [54]. It is highly active against Kasumi-1 AML cells (IC_50_ = 0.9 µM) and SkBr3 breast cancer cells (IC_50_ = 0.8 µM) and a potent apoptosis inducer by caspase-3/7 activation (IC_50_ = 1.2 µM) in MOLM13 AML cells. **7** degraded Her2 and Raf-1 in SkBr3 breast cancer cells as a consequence of Hsp70 inhibition.

The dye methylene blue (Figure 1, **8**) showed manifold biological activities, and, thus, it was also identified as an inhibitor of the Hsp70 ATPase, leading to a rapid suppression of Tau protein in neurodegenerative cell models [55]. In HeLa cervix carcinoma cells expressing poly-glutaminylated AR (AR112Q), compound **8** inhibited the Hsp70-mediated degradation of AR112Q [56]. In A375 and G361 metastatic melanoma cells, compound **8** suppressed the heat shock response (downregulation of Hsp27, Hsp70, and Hsc70), induced ROS formation, and caused glutathione depletion at a concentration of 10 µM [57]. Geldanamycin is an Hsp90 inhibitor, which increases Hsp70 expression, but compound **8** (10 µM) was able to suppress geldanamycin-induced Hsp70 expression in A375 melanoma cells. Hence, 10 µM of compound **8** also sensitized A375 melanoma cells to geldanamycin treatment, but it also sensitized these cells to treatment with etoposide and doxorubicin. Compound **8** possessed antiproliferative properties against A549 NSCLC cells and induced early apoptosis, yet it enhanced the degradation of N-terminal AR fragments as well as autophagy [58]. In mice, compound **8** inhibited benzo[a]pyrene induced lung carcinogenesis and suppressed Hsp70 as well as the tumor biomarkers ADA and LDH.

The structures of competitive and allosteric NBD binders are shown in Figure 1.

**Table 1:** Effects of NBF-targeting Hsp70 inhibitors on cancers.

Compound	Cancer Model(s)	Effects
**1** (VER-155008)	HCT-116 colon carcinoma, A549 NSCLC, pleural mesothelioma, LNCaP95 prostate carcinoma, anaplastic thyroid carcinoma, glioma (1321N1, GOS-3, U87-MG), muscle-invasive bladder cancer, osteosarcoma, multiple myeloma, acute myeloid leukemia	Selective Hsp70/Hsc70 inhibition, antiproliferative, suppression of Her2 and Raf-1, G1 cell cycle arrest, sensitization to 17-AAG and radiation, suppression of Akt and phospho-Akt, macroautophagy induction, suppression of AR-FL and ARV7, apoptosis and paraptosis induction, upregulation of miR-215 and miR-194-5p, degradation of KDMA6 and EP300, degradation of BAG1, upregulation of CHOP, suppression of VEGF release by leukemia cells, synergy effects with drugs (manumycin A, bortezomib, shikonin, 17-DMAG) and PDT [19,20,21,22,23,24,25,26,27,28,29,30,31,32,33,34,35]
**2a** (Apoptozole)	SK-OV-3 ovarian carcinoma, HCT-15 colon carcinoma, A549 NSCLC	Suppression of Hsp70-APAF-1, antiproliferative, pro-apoptotic [37]
**2b**, **2c**	HeLa cervix carcinoma, MDA-MB-231 breast carcinoma, HepG2 hepatoma, A549 NSCLC, RKO colon carcinoma, leukemia	Suppression of Hsp70-APAF-1, antiproliferative, sensitization to doxorubicin, in vivo tumor growth inhibition of A549, RKO, and HeLa xenografts, apoptosis induction, autophagy inhibition, cathepsin release [38,39,40]
**3a** (YM-1)	MCF-7 breast carcinoma, HeLa cervix carcinoma, U251 and U343 glioma, doxorubicin-resistant BT-549^r^DOX breast carcinoma	Inhibition of Hsp70-Bag3, suppression of FoxM1 and HIF1α pathways, in vivo inhibition of MCF-7 tumor growth, induction of p21, suppression of FoxM1 and surviving, sensitization of glioma to (−)-gossypol (AT-101) and ABT-737, suppression of Mcl-1, synergistic antiproliferative effects with doxorubicin [41,42,43]
**3b** (JG-98)	Triple-negative MDA-MB-231 and hormone sensitive MCF-7 breast cancer, lung cancer, miscellaneous	Inhibition of Hsp70-Bag3, FoxM1 suppression, upregulation of p21 and p27, sensitization of breast cancer to bortezomib in vivo, inhibition of TAM infiltration, sensitization of lung cancer to manumycin A [31,44,45,46,47]
**3d**, **3e**	Triple-negative breast cancer (e.g., MDA-MB-231, MDA-MB-468)	Stable, antiproliferative, tumor-selective, induction of apoptosis and autophagy, degradation of KRAS, suppression of FoxM1, survivin, HuR, and Akt [48]
**3f** (JG-231)	MCF-7 and MDA-MB-231 breast cancer	Stable, antiproliferative, inhibition of Hsp70-Bag3, degradation of Akt and HuR, tumor growth inhibition in vivo [49]
**4** (Pinaverium bromide)	A2058 melanoma	Apoptosis induction [50]
**5a** (S1g-2)	CML	Inhibition of Hsp70-Bim, apoptosis induction, suppression of Akt, Raf-1, eIF4E and RPS16 [51]
**5b** (S1g-6)	Miscellaneous	Inhibition of Hsp70-Bim, apoptosis induction, degradation of Akt and Raf-1 [52]
**6** (HS-72)	BT474, MCF-7 and SkBr3 breast carcinoma, Her2-overexpressing MMTV-*neu* spontaneous breast tumor mouse model	Selective Hsp70i inhibition, antiproliferative, Her2 and Akt degradation, tumor growth inhibition and prolonged survival in vivo [53]
**7** (YK-5)	Kasumi-1 AML, SkBr3 breast carcinoma, MOLM13 AML	Antiproliferative, apoptosis induction, Her2 and Raf-1 degradation [54]
**8** (Methylene Blue)	AR112Q-expressing HeLa cervix carcinoma, A375 and G361 melanoma, A549 NSCLC	Heat shock response suppression, ROS formation, glutathione depletion, suppressed geldanamycin-induced Hsp70, sensitization of cancer cells to geldanamycin, etoposide and doxorubicin, apoptosis induction, inhibition of lung carcinogenesis in vivo [56,57,58]

**Figure 2:**
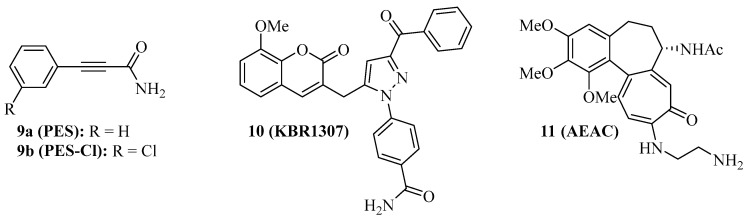
Structures of SBD-interacting Hsp70 inhibitors **9**–**11**.

Some Hsp70 inhibitors with SBD-targeting properties were described (Figure 2). Compound **9a** (Figure 2, 2-phenylethynesulfonamide, PES, pifithrin-µ) is a prominent example, which was thoroughly studied for its Hsp70-related effects in various cancers. Compound **9a** showed antiproliferative activities against various osteosarcoma, breast, and pancreatic carcinoma cell lines at IC_50_ values of 5–10 µM independent of the p53-state of the tumor cells, induced cell death independent of caspase activation, and led to dysfunctional autophagy by the formation of p62-oligomers/aggregates [59]. It decreased the interaction of Hsp70 with APAF1, p53, and the co-chaperones Hsp40, CHIP, and BAG-1M and suppressed NF-κB signaling and activity. In vivo, compound **9a** (40 mg/kg, i.p., every five days for 30 days) blocked Myc-based lymphopathogenesis and led to prolonged survival in Eµ-Myc transgenic mice. Compound **9a** exhibited considerable antiproliferative activity against acute leukemia (AML and ALL) cells (IC_50_ = 2.5–12.7 µM), induced apoptosis in these cells by caspase activation, and led to a degradation of Akt and ERK1/2 [60]. In addition, compound **9a** sensitized acute leukemia cells to treatment with cytarabine (an antimetabolite), 17-AAG (a Hsp90 inhibitor), vorinostat (a HDAC inhibitor), and sorafenib (a RTK inhibitor). In primary effusion lymphoma (PEL), compound **9a** exerted cytotoxic effects on BC3 and BCBL1 cells by apoptosis and another cell death mechanism, which was associated with immunogenic activity such as activation of dendritic cells [61]. Compound **9a** increased lysosome permeabilization and cathepsin D release in PEL cells, accompanied by Bid cleavage, outer mitochondrial depolarization, and AIF translocation to the nucleus. Moreover, compound **9a** sensitized PEL cells to bortezomib treatment. The combination of compound **9a** with DNA-targeting platinum complexes such as cisplatin and oxaliplatin also revealed synergy effects in HT-29 colon carcinoma and PC-3 prostate carcinoma cells [62]. In prostate cancer cells, compound **9a** also increased the antitumor effects of hyperthermia (HT, 43 °C), best when given immediately before HT started, which was accompanied by upregulation of p21 and suppression of c-Myc and cyclin D1 [63]. The combination of compound **9a** (100 µg in 50 µL) and HT (43 °C for 1 h, twice on days 0 and 4), led to significant PC-3 prostate carcinoma xenograft growth inhibition. Its modified analog compound **9b** (Figure 2, PES-Cl) was antiproliferative against a panel of BRAF-V600E mutant melanoma (IC_50_ values between 2–5 µM, while inactive against melanocytes) and showed higher antiproliferative activity than compound **9a** against SkBr3 breast carcinoma, FaDu head and neck squamous cell carcinoma, and H1299 lung adenocarcinoma cells [64]. The cytotoxic activity of compound **9b** is based on apoptosis induction (caspase activation) and inhibition of autophagy (p62 accumulation), while the HeLa cell cycle was arrested in the G2-M phase by compounds **9a** and **9b** associated with cyclin B1 degradation. At doses of 20 mg/kg (i.p., once per week), compound **9b** led to a much higher survival rate of Eµ-Myc mice (71.4% survival) than compound **9a** (35% survival) after 210 days. Compound **9b** also induced apoptosis in A375 melanoma cells, accompanied by Her2 degradation in these cells [65]. In contrast to compounds **1** and **3c**, only compound **9b** led to G2-M arrest in H1299 and A375 cells based on cyclin B1 degradation.

Compound **9a** does not interact with Hsp70 when no nucleotide is bound to the protein. The coumarin-pyrazole hybrid, compound **10** (Figure 2, KBR1307) was designed, which binds Hsp70 in the presence and absence of nucleotides [66]. Both compounds **9a** and **10** reduce the activity of Hsp70 ATPase significantly, but compound **10** was more active against MCF-7 cells than compound **9a**. Similar coumarin-thiazole hybrids were described by the same group before as binders to the C-terminus of Hsp70 with activity against DLD-1 colon carcinoma and HepG2 hepatoma cells [67].

The screening of the InterBioScreen compound library for Hsp70 inhibitors revealed that the semi-synthetic colchicine derivative, compound **11** (Figure 2, *N*-aminoethylaminocolchicine, AEAC), interferes with substrate binding and refolding functions of Hsp70, based on a nanomolar affinity for Hsp70 (K_D_ = 149 nM) [68]. Although the antiproliferative and cytotoxic activities of compound **11** are low, it sensitized C6 rat glioblastoma and B16 mouse melanoma cells to doxorubicin treatment. The combination of compound **11** (2 mg/kg) and doxorubicin (1 mg/kg) inhibited in vivo B16 tumor growth by 71% and increased the lifespan of treated mice by ca. 15 days when compared with untreated mice.

The structures of SBD-interacting compounds are shown in Figure 2. Table 2 summarizes the anticancer effects of the described SBD-domain interacting Hsp70 inhibitors.

### 2.2. Grp78 Inhibitors

**Figure 3:**
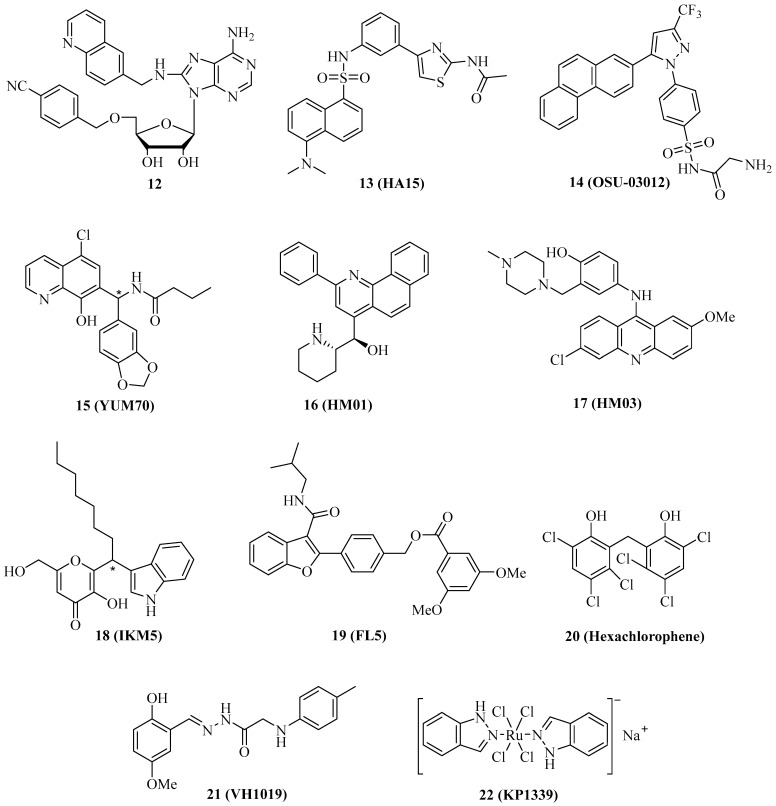
Structures of Grp78 inhibitors **12**–**22**. *: chiral center or asymmetric carbon atom.

The specific targeting of organelle-specific Hsp70 isoform proteins such as Grp78 (ER) and mortalin (mitochondria) has become a valuable strategy to combat cancer. Grp78 is the “master protein” of UPR (unfolded protein response) and redirects misfolded polypeptides for degradation or refolding [14]. Some of the already described Hsp70 inhibitors also inhibit Grp78. For instance, compound **1** is likewise a Grp78 inhibitor. In OSA cells, compound **1** inhibited Grp78 in addition to Hsp70, followed by antiproliferative and proapoptotic effects [29]. In MCF-7 and MDA-MB-231 breast cancer cells, Grp78 was upregulated upon tamoxifen treatment, leading to resistance; however, Grp78-inhibitory **1** enhanced apoptosis induction by tamoxifen in these cells, accompanied by suppression of tamoxifen-induced phosphor-GSK-3β, which is a downstream factor of Akt signaling [69]. The quinoline analog **12** (Figure 3) of compound **1** was slightly more active against and selective for Grp78 (K_D_ = 0.6 µM for Grp78, 0.3 µM for Hsp70) than compound **1** (K_D_ = 0.8 µM for Grp78, 0.1 µM for Hsp70), however, compound **12** showed no antiproliferative activity against HCT-116 colon cancer cells in contrast to compound **1** (GI_50_ = 5.0 µM) [70]. The absence of antiproliferative activity in compound **12** was explained by the detrimental physicochemical properties of this compound, which need to be improved in order to obtain a valuable adenosine-based Grp78-selective inhibitor in the future.

The thiazole benzenesulfonamide **13** (Figure 3, HA15) was identified as a Grp78 inhibitor that enhanced ER stress associated with autophagy and apoptosis, leading to cell death in melanoma cells, in particular in BRAF-mutant cells, at a concentration of 10 µM [71]. In vivo, compound **13** (0.7 mg/mouse/day for 2 weeks, i.p.) inhibited A375 melanoma growth without causing side effects such as mouse weight loss and liver damage. Grp78 was found to be upregulated in lung cancers, and treatment with compound **13** led to antiproliferative effects on A549 lung cancer cells as well as to apoptosis induction, autophagy, and increased ER stress [72]. The GRp78 inhibitor **13** suppressed KRAS expression and revealed antiproliferative and pro-apoptotic activities in various KRAS-mutant cancer cell lines (A427 lung adenocarcinoma, H460 non-small cell lung carcinoma, HCT-116 and LS180 colon carcinomas, PANC-1 and CFPAC-1 pancreatic ductal adenocarcinomas), and induced apoptosis in A427, HCT-116, and PANC-1 cells by caspase activation [73]. Compound **13** also suppressed cell proliferation and steroidogenesis in adrenocortical carcinoma (ACC) cells, and showed synergy effects in combination with the approved drug mitotane, which is also an activator of ER stress [74].

In carboplatin-resistant canine osteosarcoma cells (HMPOS-2.5R and HMPOS-10R), compound **13** and the atypical Grp78 inhibitor **14** (Figure 3, the celecoxib derivative OSU-03012, which binds directly to the Grp78 ATPase domain) still showed considerable activity (EC_50_ = 1.9–3.5 µM for **13** and 5.3–8.5 µM for **14**), while the Hsp70 inhibitor **1** was distinctly less active against these resistant cells (EC_50_ = 25–30 µM) than against the parent HMPOS cells (EC_50_ = 1.8 µM) [75,76]. Compound **14** reduced the expression of Bag2, and treatment with compound **14**, especially in combination with the PDE5 inhibitor sildenafil, formed toxic autophagosomes that inducing cell death in GBM5 and GBM12 glioblastoma cells [77]. Similar effects were observed for the multi-kinase inhibitor sorafenib, which is an approved anticancer drug and also able to bind to the N-terminal domain of Grp78.

Analogously to compound **13**, the hydroxyquinoline derivative **15** (Figure 3, YUM70) was able to downregulate KRAS, leading to antiproliferative and pro-apoptotic effects on KRAS-mutant cancer cells [73]. Initially, compound **15** was identified as an antiproliferative and caspase-dependent pro-apoptotic compound in pancreas cancer cells (IC_50_ = 2.8 µM for MiaPaCa-2, 4.5 µM for PANC-1, and 9.6 µM for BxPC-3 cells) based on Grp78 inhibition (by binding to the SBD) followed by ER stress via eIF2α phosphorylation as well as AT4 and CHOP activation [78]. Synergy effects of compound **15** on MiaPaCa-2 cells were observed in combination with vorinostat or topotecan. In the MiaPaCa-2 xenograft model, compound **15** (30 mg/kg, 5 days a week for 7 weeks, i.p.) inhibited tumor growth and caused weight loss in treated mice.

The *N*-heteroaromatic compounds **16** (Figure 3, HM01) and **17** (Figure 3, HM03) were identified as Grp78 inhibitor hits from the virtual screening of an NCI diversity set, and they showed considerable affinity to the substrate-binding channel of Grp78 [79]. Both compounds exerted moderate antiproliferative activity against HCT-116 colon cancer cells (IC_50_ between 10 and 25 µM) but may serve as lead compounds for the design of more potent Grp78 inhibitors.

The indolylkojyl derivative **18** (Figure 3, IKM5) was prepared by a simple three-component reaction and identified as a potent Grp78 inhibitor (K_i_ = 1.4 µM), which showed high antiproliferative activities against a panel of breast cancer cell lines (IC_50_ = 0.15 µM for MCF-7, 0.21 µM for MDA-MB-231, 0.54 µM for MDA-MB-468, and 3.5 µM for BT474 cells) [80]. Compound **18** induced TIMP-1 by blocking its interaction with Grp78, suppressed EMT markers such as MMP-2, Twist1, and vimentin, and upregulated the Par-4 tumor suppressor, which controls NF-κB signaling. Compound **18** increased the activity of doxorubicin against invasive breast cancer cells. In vivo, compound **18** (30 mg/kg, b.w.) inhibited 4T1 breast tumor growth by 79.2% and lung metastasis formation by 84.5%.

The benzofuran derivative **19** (Figure 3, FL5) is a strong binder and stabilizer of Grp78 (T_m_ increase > 2 °C), which was associated with its anticancer and antiangiogenic activities against renal cell carcinoma (RCC) cells (10 µM of **19** led to 50% cell death) and HUVECs (EC_50_ = 1.5 µM), while it was inactive against mouse fibroblasts [81]. Docking studies showed that compound **19** does not interfere with the ATPase activity of Grp78.

A high-throughput substrate binding assay led to the identification of the disinfectant hexachlorophene (Figure 3, **20**) as a Grp78 inhibitor binding to the SBD [82]. Compound **20** was cytotoxic against HCT-116 colon carcinoma cells (CC_50_ = 3.4 µM) and induced apoptosis and autophagy, as well as an unfolded protein response associated with upregulated ATF4, XBP1s, and CHOP.

In silico methods for the design of new Grp78 inhibitors led to the identification of **21** (Figure 3, VH1019) with antiproliferative activity against MCF-7 breast cancer cells (IC_50_ = 12.7 µM) [83]. It is noteworthy that compound **21** mimicked ATP in its binding to Grp78.

In addition to organic compounds, metal complexes can be a valuable source of Grp78 inhibitors. The anionic ruthenium complex **22** (Figure 3, KP1339, BOLD-100) is an efficient Grp78 suppressor that has already demonstrated promising anticancer activity in phase 1 clinical trials, both as compound **22** and in its benzindazolium salt form, KP1019 [84]. A phase 1b/2 clinical trial with the metallodrug compound **22** for patients suffering from gastrointestinal cancer is ongoing (NCT04421820). Mechanistic studies revealed apoptosis induction in sensitive cancer cell lines by activation of caspase-8, which was associated with disruption of the ER upon suppression of key chaperones, leading to the degradation of vital proteins [85]. In contrast, cancer cells with a low response to complex compound **22**-induced G2 cell cycle arrest may serve as a general hint at cancers resistant to Grp78 inhibitors. Interactions with the ribosomal proteins RPL10 and RPL24 and with the transcription factor GTF2I were identified in HCT-116 colon carcinoma cells and associated with ribosomal disturbance and ER stress induction [86]. Grp78 is upregulated in asbestos-associated pleural mesothelioma, and, thus, the activity of **22** against this cancer was evaluated. Complex compound **22** was cytotoxic against mesothelioma cells (EC_50_ = 71 µM for REN and 90 µM for MM98 cells), inhibited REN cell colony formation at a concentration of 100 µM, induced apoptosis in REN cells by activation of caspase-3/7 and caspase-8, increased ROS formation and cytosolic Ca^2+^ levels, and suppressed Grp78 expression in REN cells [87]. CHOP and XPB1 expression were upregulated by compound **22** in REN cells. Sensitive HCT-116 colon carcinoma (IC_50_ = 76 µM) and Capan1 pancreatic carcinoma cells (IC_50_ = 40 µM) were used to study cell-based resistance to treatment with compound **22** [88]. Increased glucose uptake and upregulated glycolysis were observed in sensitive cancer cells upon treatment, but especially in the resistant cell lines HCTR and CapanR obtained from HCT-116 and Capan1 cells, respectively, upon exposure to compound **22**. However, this specific mechanism made the resistant tumor cells highly vulnerable to the treatment with the glycolysis inhibitor and ER stress inducer 2-dexyglucose, which led to synergy effects of combinations of compound **22** with 2-deoxyglucose in resistant HCTR cells.

The structures of the described Grp78 inhibitors are shown in Figure 3, and their activities are summarized in Table 3.

### 2.3. Mortalin Inhibitors

**Figure 4:**
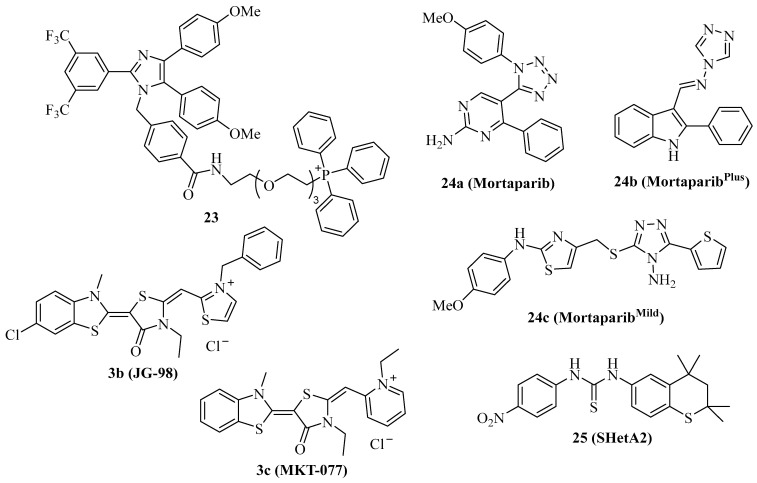
Structures of mortalin inhibitors **3b**, **3c**, **23**–**25**.

Mortalin is the mitochondrial Hsp70 isoform with an N-terminal mitochondrial localization motif and has become an important target for cancer therapy [15]. Due to its preferred localization in mitochondria, mortalin is involved in the regulation of mitochondrial metabolism and of key tumor factors such as p53, PI3K/AKT, Raf/MEK/ERK, and JAK/STAT pathways [15,89]. The triphenylphosphonium (TPP) moiety was described as a mitochondria-targeting device, and, thus, compound **2b** was conjugated with TPP in order to obtain the mortalin-targeting apoptozole conjugate compound **23** (Figure 4) [40]. Compound **23** showed higher antiproliferative activity against a panel of 20 cancer cell lines (IC_50_ = 0.5–1.5 µM) than compound **2b** and induced caspase-dependent apoptosis in HeLa cells by blocking the interaction of p53 with mortalin in the mitochondria, followed by Bak-mediated mitochondrial outer membrane permeabilization.

Cationic Hsp70 inhibitors such as compound **3b** (Figure 4, JG-98) were identified as mortalin inhibitors with pronounced activity against proteasome inhibitor-resistant MM cells associated with 55S mitoribosome degradation [90]. Compound **3c** (Figure 4, MKT-077) overlaps with the p53-binding region of mortalin, releasing active p53 followed by upregulation of p21 in treated cancer cells [91,92]. In addition, compound **3c** sensitized K562 leukemia cells to complement-mediated lysis by inhibition of mortalin and its interaction with the C9 complement protein [93]. Compound **3c** was identified already in the 1990s as a mitochondria-targeting anticancer compound with preference for ras-associated cancers, and entered clinical trials after promising in vivo results [94,95,96,97]. However, complications such as renal toxicity prevented the approval of compound **3c** as an anticancer drug [98].

The tetrazole derivative **24a** (Figure 4, mortaparib) is a relatively new dual mortalin and PARP1 inhibitor that was developed in 2019 [99]. Compound **24a** led to the activation and nuclear accumulation of p53 by inhibiting mortalin. In addition, PARP1 was downregulated by compound **24a,** followed by increased double-strand breaks and apoptosis induction in HeLa cervix carcinoma and SKOV-3 ovarian cancer cells. In vivo, compound **24a** (20 mg/kg i.p.) was well tolerated, inhibited the tumor growth of SKOV-3 xenografts, and suppressed the formation of metastases in the lungs and kidneys. Based on these promising anticancer activities of compound **24a**, the same group developed further mortaparib derivatives. The 1,2,4-triazole **24b** (Figure 4, mortaparib^Plus^) blocked the interaction of p53 with mortalin, leading to p53 activation and suppression of PARP1 and CARF in HCT-116 cells [100]. Compound **24b** induced apoptosis and activated p21 in HCT-116 (p53 wildtype) and DLD-1 (p53^S241F^) colon carcinoma cells in p53-dependent and –independent ways. In contrast to colon cancer cells, in breast cancer cells, compound **24b** upregulated only p21 in the p53-wildtype MCF-7 cells, while T47D cells (p53^L194F^) treated with compound **24b** showed no p21 changes but activation of PARP1, albeit compound **24b** inhibited the mortalin-p53 interaction in both cell lines [101]. More recently, compound **24c** (Figure 4, mortaparib^mild^) was identified as an inhibitor of PARP1 and mortalin-p53 interaction in HCT-116 cells, however, at higher concentrations than compounds **24a** and **24c**, thus leading to the attribute “mild” for compound **24c** [102].

The thiochromane derivative **25** (Figure 4, SHetA2) is an orally bioavailable mortalin inhibitor that blocks the interaction of mortalin with p53 in ovarian cancer cells [103]. Due to its promising chemo-preventive and selective anticancer activities, including apoptosis induction, compound **25** has entered clinical phase 1 studies for patients with advanced/recurrent cervical, endometrial, or ovarian cancer [15]. Since mutant p53 led to drug resistance upon treatment with compound **25**, strategies to overcome this resistance are sought. A promising strategy is the combination of compound **25** with the p53 reactivator PRIMA-1^Met^, which was studied in a panel of ovarian cancer cell lines [104]. PRIMA-1^Met^ reduced resistance to compound **25** and exhibited synergy effects in combination with compound **25** in p53-mutant and p53-wildtype ovarian cancer cells, accompanied by caspase activation, increased ROS formation, and reduced ATP. The combination of compound **25** (60 mg/kg by gavage every day for 2 weeks, then every second day for 3 weeks) and PRIMA-1^Met^ (10 mg/kg, i.p. every other day) inhibited MESOV tumor growth (tumor free rate of 67%) in an additive way without toxicity to the liver and kidneys.

The structures of the described small-molecule mortalin inhibitors are shown in Figure 4. Their anticancer activities are summarized in Table 4.

## 3. Modulators of Co-Chaperone Hsp40

**Figure 5:**
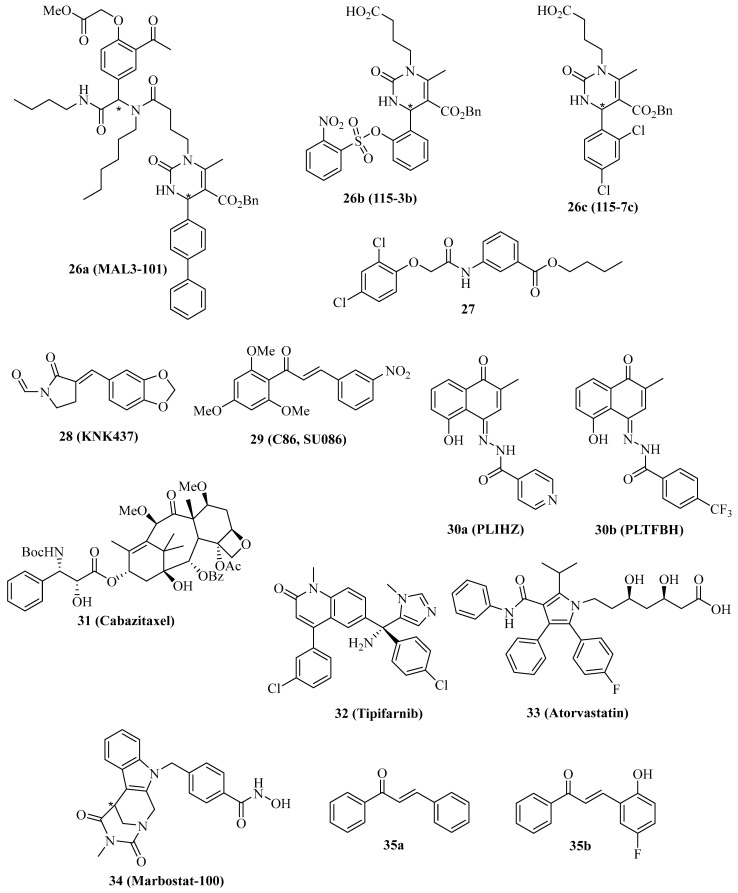
Structures of Hsp40 modulators **26**–**35**. *: chiral center or asymmetric carbon atom.

The co-chaperones of the Hsp40 family are vital for the ATPase activity of Hsp70 proteins, and modulation of Hsp40 activity has tremendous effects on the Hsp70 network of protein interaction and integrity. The natural Hsp70 ATPase stimulator 15-deoxyspergualin served as a lead compound for the design of the uracil derivative NSC-630668-R/1, which inhibits endogenous and Hsp40-induced ATPase activity of Hsp70 [105,106]. Further synthetic derivatives were prepared using the straightforward Biginelli multicomponent reaction, leading to the identification of **26a** (Figure 5, MAL3-101), which specifically blocks Hsp70 ATPase in an allosteric way by inhibition of the Hsp40 co-chaperone protein TAg [107]. In this way, the strategy to target the Hsp70/Hsp40 axis as a possible treatment for cancer diseases was established. In contrast, the synthetic small-molecule dihydropyrimidine compounds **26b** (Figure 5, 115-3b) and **26c** (Figure 5, 115-7c), which lack the amide side chain of **26a**, stimulated protein folding by the bacterial DnaK (Hsp70) chaperone in the presence of the DnaJ (Hsp40) and GrpE co-chaperones, while compound **26a** inhibited protein folding in this assay as expected with an EC_50_ value of 3.2 µM [108,109].

Compound **26a** showed various anticancer properties, e.g., antiproliferative activity against Merkel cell carcinoma (MCC) accompanied by apoptosis induction [110]. In addition, compound **26a** (40 mg/kg i.p. every other day) inhibited WaGa MCC growth in mice, again associated with apoptosis induction in vivo. Moreover, compound **26a** (10 µM) showed antiproliferative activity against T24 and SW780 MIBC cells, and the combination of compound **26a** with the Hsp70 inhibitor **1** or with the Hsp90 inhibitor STA-9090 led to synergistic antiproliferative activity against MIBC cells, which were resistant to compound **26a** alone [111]. RMS13 rhabdomyosarcoma cells were also sensitive to treatment with **26a** based on the induction of UPR and apoptosis; however, dose-escalation led to the isolation of a resistant RMS13-R cell line with upregulated autophagy and an ER-associated degradation pathway [112]. Inhibition of autophagy restored the sensitivity to **26a** in the RMS13-R cells.

The phenoxy-*N*-arylacetamide **27** (Figure 5) was found to inhibit Hsp70 by direct binding to the co-chaperone DnaJ (IC_50_ = 0.13 µM) [113].

In 2000, the synthetic lactam derivative **28** (Figure 5, KNK437) was identified as a suppressor of heat-induced Hsp70 and Hsp40 in COLO 320DM human colon cancer cells, whose effect was more pronounced than for the known natural Hsp inhibitor quercetin [114]. In vivo, compound **28** (200 mg/kg i.p.) showed synergy effects in combination with heat treatment (44 °C) in mice bearing SCC VII squamous cell carcinomas [115]. At a non-toxic concentration of 300 µM, compound **28** inhibited colony formation of p53-mutant SAS/mp53 human squamous cell carcinoma cells at a temperature of 42 °C [116]. This inhibitory effect of compound **28** in combination with heat was stronger than in the p53-wildtype SAS/neo cells and accompanied by the induction of apoptosis at a reduced concentration (100 µM). Compound **28** (100 µM) also inhibited heat-induced (44 °C) transcription-activating histone H3-Lys4 methylation both in thermoresistant HSC4 and in thermosensitive KB oral squamous cell carcinoma cells, which was associated with Hsp70 suppression by compound **28** [117]. In immortalized Cos-1 cells, 100 µM of compound **28** inhibited mTOR and S6K phosphorylation and suppressed mTORC1 activity, leading to apoptosis induction [118]. Compound **28** (50 µM) sensitized MDA-MB-231 breast cancer cells to ionizing radiation by mechanisms independent from Hsp suppression [119]. Instead, hypoxia-related AKT and HIF-1α survival pathways were inhibited by compound **28,** which were interesting off-targets of this compound. The Hsp40 protein DNAJA1 was upregulated in colorectal cancer cells, but treatment with compound **28** strongly inhibited the level of the Hsp40 protein DNAJA1 in SW480 (IC_50_ = 24.7 µM), RKO (IC_50_ = 25.5 µM), LOVO (IC_50_ = 56.0 µM) and SW620 cells (IC_50_ = 48.3 µM), while no effects on Hsp70 and Hsp90 were found [120]. DNAJA1-overexpressing SW480 and SW620 cells exhibited much faster and stronger tumor growth in vivo, which was inhibited by treatment with compound **28** (20 mg/kg i.p. for 20 days). The combination of 5-FU/L-OHP with compound **28** (20 mg/kg i.p.) efficiently suppressed liver metastasis formation in DNAJA1-overexpressing SW480 colorectal tumors. Mechanistically, compound **28** suppressed CDC45 and upregulated ubiquitin in DNAJA1-overexpressing SW480 cells as a consequence of its strong DNAJA1 downregulating activity.

The chalcone compound **29** (Figure 5, C86/SU086) was initially identified as a synthetic xanthohumol analog with antiproliferative activity against HeLa cervix carcinoma cells (IC_50_ = 1.4 µM), pro-apoptotic activity by caspase-3 activation, and thioredoxin reductase inhibitory activity (IC_50_ = 3.5 µM) followed by induction of ROS formation in treated HeLa cells [121]. The effects of compound **29** on castration-resistant prostate cancer (CRPC) were studied in more detail, revealing strong antiproliferative activity against 22Rv1 CRPC cells (IC_50_ between 1 and 2.5 µM) and a suppression of FL-AR/ARv7 signaling based on enhanced degradation of FL-AR and ARv7 in 22Rv1 CRPC cells treated with compound **29** (10 µM) [122]. Experiments using biotinylated 29 confirmed a direct binding as a pan-Hsp40/DNAJ inhibitor, which interacts with DNAJA, DNAJB, and DNAJC proteins likely via the J domains of the Hsp40 proteins. In addition, compound **29** is bound to Hsp40 in complexes with AR and Arv7 and with Hsp70/Bag3/CHIP. Similar effects were observed for the Hsp70 inhibitor **3b** (JG98, IC_50_ = 0.4–0.5 µM for 22Rv1 cells, destabilization of FL-AR/ARv7 proteins). Both compounds **29** (15 mg/kg, i.v. 3 x per week) and **3f** (8 mg/kg, i.p. every other day) inhibited the growth of 22Rv1 CRPC xenografts in mice; however, the combination of both drug candidates surpassed the antitumor activity of the single compounds. More recently, compound **29** was also identified as a Hsp90 inhibitor in prostate cancer cells, which interferes with tumor cell glycolysis [123]. In C4-2 prostate cancer xenografts, compound **29** (50 mg/kg/day i.p.) inhibited tumor growth similar to the approved prostate cancer drugs enzalutamide (10 mg/kg/day p.o.) and abiraterone (200 mg/kg/day p.o.). Yet, the combination of compound **29** with either enzalutamide or abiraterone led to synergistic and additive tumor growth inhibitory effects, respectively.

The semi-synthetic hydrazones **30a** (Figure 5, PLIHZ) and **30b** (Figure 5, PLTFBH), which were made of the natural naphthoquinone plumbagin, were identified as Hsp40 inhibitors by molecular docking [124]. Compounds **30a** and **30b** revealed antiproliferative activities against HN31 pharyngeal squamous cell carcinoma cells (IC_50_ = 1.2 µM for **30a** and 0.6 µM for **30b**) and suppressed DNAJA1 and conformational mutant p53 levels in HN31 cells, accompanied by suppression of tumor cell migration based on downregulation of active Cdc42 and Rac. No effects were observed for wild-type p53 or DNA-contact mutant p53 proteins, indicating a selective action against conformational mutant p53. In addition to DNAJA1, compound **30b** showed distinct inhibitory activities against DNAJA2, DNAJA3, DNAJB1, DNAJB12, and DNAJC3.

The semi-synthetic 3rd generation taxane derivative cabazitaxel (Figure 5, **31**) was able to inhibit LNCaP and PC-3 prostate cancer cell proliferation and suppress Hsp40, HOP, and AR in prostate cancer cells at very low doses (0.3 nM) [125].

The inhibition of farnesylation of HDJ-2 Hsp40 proteins has become a reasonable marker for the clinical outcome of anticancer active farnesyl transferase inhibitors [126,127,128]. Farnesyl transferase inhibitors such as tipifarnib (Figure 5, **32**) entered clinical trials for various cancer diseases because they inhibit the farnesylation of Ras proteins, leading to their inactivation [129,130]. The radio-sensitizing effect on SF763 glioblastoma cells treated with compound **32** was associated with a suppression of the radio-induced translocation of HDJ-2 by compound **32** based on reduced levels of farnesylated HDJ-2 [131]. In addition, compound **32** inhibited U87 and SF763 glioblastoma cell proliferation (IC_50_ = 3.1 µM for U87 and 1.9 µM for SF763) and led to G2/M arrest in these cells based on p21 induction. The HMG-CoA reductase inhibitory statin drug **33** (Figure 5, atorvastatin), which is applied for the treatment of cardiovascular disease based on its cholesterol depletion activity, was also studied for its inhibition of DNAJA1 farnesylation in pancreatic cancer cells expressing wild-type or mutant p53 proteins [132]. Compound **33** induced apoptosis, upregulated p21, and degraded mutant p53 (R172H) and cyclin D1 in PO3 cells based on the suppression of farnesylated DNAJA1. In addition, compound **33** suppressed mutant p53 in SU 86.86 (G245S), BXPC-3 (Y220C), and Pan 10.05 (I255N) pancreatic cancer cells, while mutant p53 levels in MIA-PaCa-2 (R248W) and PANC-1 (R273H) were not affected by compound **33**. Nuclear translocation of mutant p53 was inhibited in PO-3 cells by compound **33**, which also suppressed PO-3 cell migration and invasion.

Selective inhibition of cytoplasmic HDAC6 by the HDAC6 inhibitor **34** (Figure 5, marbostat) led to MYC degradation and apoptosis in MYC-overexpressing B-cell lymphoma cells. It was shown that HDAC6 inhibition by compound **34** led to hyperacetylation of tubulin followed by enhanced binding of DNAJA3 to hyperacetylated tubulin in the cytoplasm of B-cell lymphoma cells, which led to enhanced Myc degradation [133].

In contrast to the Hsp40-inhibiting chalcone **29**, the trans-chalcone **35a** (50 µM) and its 5-fluoro-2-hydroxy derivative **35b** (10 µM) activated Hsp40 and p53 expression in U2OS osteosarcoma cells, accompanied by suppression of CRM1 (Figure 5) [134].

The structures of the described small-molecule Hsp40 modulators are shown in Figure 5. Their anticancer activities are summarized in Table 5.

## 4. Discussion

**Figure 6:**
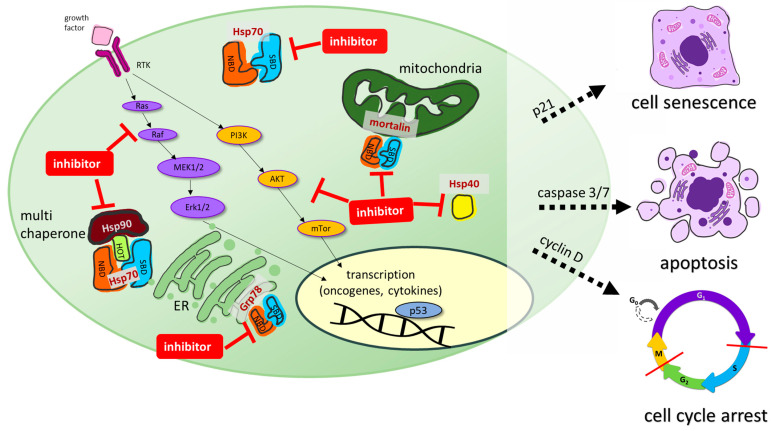
Cellular effects of Hsp70 isoforms and their inhibition by synthetic small molecules. Hsp70 is composed of two distinct domains, a 40 kDa N-terminal nucleotide-binding domain (NBD) that regulates client association and a 25 kDa C-terminal substrate-binding domain (SBD). Hsp70 is found in the cytosol with multicellular functions. Together with Hsp90 it acts as a multi-chaperone complex to modulate heat stress responses. Organelle-specific Hsp70 isoforms such as Grp78 in the endoplasmic reticulum or mortalin in mitochondria were identified as promising anticancer drug targets. Inhibition of these multicellular processes by (semi-)synthetic inhibitors (red lines) leads to cell senescence, apoptosis and cell cycle arrest in various cancers.

The design and development of inhibitors of the Hsp70-Hsp40 axis is a prospering field of research. Numerous Hsp70 inhibitors were described, and the number of Hsp40 inhibitors and modulators is also growing. Hsp70 is essential for the survival of proliferating cancer cells, where it is often overexpressed, while it is more or less dispensable for non-transformed cells, making it an excellent anticancer drug target for tumor-selective drug candidates. Hsp70 is composed of two distinct domains, a 40 kDa N-terminal nucleotide-binding domain (NBD) that regulates client association and a 25 kDa C-terminal substrate-binding domain (SBD). Hsp70 is found in the cytosol and possesses multiple cellular functions. Together with Hsp90, it acts as a multi chaperone complex to modulate cellularly-protective heat stress responses. Organelle-specific Hsp70 isoforms such as Grp78 in the endoplasmic reticulum or mortalin in the mitochondria were also identified as promising anticancer drug targets for Hsp70 inhibitors [14,15]. Hsp40 is another member of the Hsp family and functions as a client protein of Hsp70. It is a small co-chaperone of Hsp70 that prevents the aggregation of unfolded polypeptides and proteins before transferring them to Hsp70 [17]. Hsp40 also binds to the nucleotide-binding domain of Hsp70, thereby increasing the ATPase activity of Hsp70. Moreover, Hsp70 is also involved in modulating receptor tyrosine kinase (RTK) signaling pathway activity, such as the Ras/Raf-MAPK or the AKT pathway. Synthetic Hsp70 inhibitors can interact with the N-terminal NBD or the C-terminal SBD of Hsp70. Some inhibitors also interfere with Hsp40 or act on organelle-specific Hsp70 isoforms such as Grp78 (ER) or mortalin (mitochondria), leading to pronounced anticancer effects such as apoptosis, cell cycle arrest, or cellular senescence (Figure 6) [14,15].

These effects are often synergistic in combination with well-established Hsp90 inhibitors, and have the potential to overcome the drawbacks of single Hsp90 inhibition in cancer therapy. A conjugate of 17-AAG with apoptozole (**2c**) designed as a dual Hsp70/Hsp90 inhibitor was superior to Hsp70 inhibitor **2b** in terms of anticancer activity [39,40]. Synthetic efforts led to promising compounds with intrinsic dual or multimodal activities, such as dual mortalin/PARP1 inhibition, which are pointing the way to more efficient anticancer drug candidates [99,100,101].

Synthetic conjugate strategies also led to subcellular isoform targeting by attachment of a mitochondria-specific triphenylphosphinium moiety to the apoptozole scaffold in the selective mortalin inhibitor **23** [40]. It is noteworthy that mitochondria-targeting cationic dyes such as rhodacyanines (**3a**–**f**) and methylene blue (**8**) were identified as Hsp70 inhibitors, which were initially developed for other applications than cancer therapy. Methylene blue was the first synthetic drug applied for the treatment of malaria and the lead compound for the development of tricyclic anti-depressant drugs [135]. Meanwhile, several rhodacyanine derivatives have also been investigated as antiprotozoal agents [136]. Thus, the repurposing of drugs appears promising and is not a one-way road. As far as drug repurposing is concerned, the disinfectant hexachlorophene (**20**) and the spasmolytic drug pinaverium bromide (**4**) are further interesting examples of Hsp70 inhibitory activity [50,82]. In contrast to the cationic mitochondria-targeting compounds with preference for mortalin binding, the anionic ruthenium complex **22** (KP1339) is an efficient Grp78 suppressor [85,86,87,88]. However, the exact anticancer mechanisms of action of such pleiotropic ruthenium complexes are only partially understood. In terms of Grp78 isoform selectivity, the modification of compounds **1** through **12** already showed a simple way to improve selectivity for Grp78 [70].

From a chemical point of view, the (semi-)synthetic Hsp70 and Hsp40 inhibitors described in this review are structurally heterogenic and comprise various compound classes. While natural Hsp70 and Hsp40 inhibitors were largely excluded from this review for clarity’s sake, some promising chemically modified natural products (**11**, **30a**, **30b**) and synthetic surrogates (**26a**, **29**) were mentioned. In terms of Hsp40 protein targeting, structurally related synthetic compounds can have opposite effects on their target. While **26a** inhibited Hsp40, its analogs compounds, **26b** and **26c**, activated Hsp40 [107,108,109,110,111,112]. In addition, chalcone **29** inhibited Hsp40, while chalcones **35a** and **35b** activated Hsp40 [121,122,123,134]. Interestingly, in the Chalcone case, both effects led to anticancer activity. Posttranslational modifications of Hsp40 (farnesylation) and its binding partners (acetylated tubulin) were also described as targetable Hsp40-modulatory mechanisms [125,131,132,133]. In this way, potential combination partners such as farnesyltransferase inhibitors and HDAC6 inhibitors emerged as modulators of the Hsp70-Hsp40 axis, which might be considered for future in vivo experiments.

As mentioned above, the combination of Hsp70 and/or Hsp40 inhibitors with various approved anticancer drugs revealed promising antitumor effects both in vitro and in vivo, suggesting suitable therapy regimens for new clinical trials. The Hsp70 inhibitors **3c** and **22** (in its benzindazolium form, KP1019) already underwent early-stage clinical trials decades ago, even before their Hsp70 inhibitory activity was discovered [84,94]. Meanwhile, compound **22** experienced a revival and is currently in phase 1b/2 clinical trials (NCT04421820) in combination with FOLFOX (folinic acid, 5-fluorouracil, and oxaliplatin) for the treatment of advanced gastrointestinal tumors. However, with various new inhibitors and optimized analogs being available now, more valuable outcomes can be expected for Hsp70 and Hsp40 inhibitors from future clinical studies when designed and conducted properly.

Hsp40 is another member of the Hsp protein family and functions as a client protein of Hsp70. It is a small co-chaperone of Hsp70 that prevents the aggregation of unfolded polypeptides and proteins before transferring them to Hsp70. Hsp40 also binds to the nucleotide-binding domain (NBD) of Hsp70, thereby increasing the ATPase activity of Hsp70. Moreover, Hsp70 is also involved in modulating receptor tyrosine kinase (RTK) signaling pathway activity, such as the Ras/Raf-MAPK or the AKT pathway.

Synthetic Hsp70 inhibitors can act at the N-terminal (NBD) or substrate-binding C-terminal (SBD) binding domains of Hsp70. Some inhibitors also interfere with Hsp40 or act on organelle specific Hsp isoforms such as Grp78 (ER) or mortalin (mitochondria), leading to pronounced anticancer effects such as apoptosis, cell cycle arrest, or cellular senescence.

## Figures and Tables

**Table 2:** SBD-interacting Hsp70 inhibitors and their effects on cancer model(s).

Compound	Cancer Model(s)	Effects
**9a** (PES)	Colon, breast, prostate and pancreas carcinoma, osteosarcoma, lymphoma, acute leukemia	Antiprolierative independent from p53-state, caspase activation, dysfunctional autophagy, prolonged survival of Eµ-Myc mice, NF-κB suppression, degradation of Akt and ERK1/2, immunogenic activity, sensitization of cancers to drugs and hyperthermia [59,60,61,62,63]
**9b** (PES-Cl)	BRAF-V600E mutant melanoma, SkBr3 breast carcinoma, FaDu head and neck squamous cell carcinoma, H1299 lung adenocarcinoma, lymphoma	More antiproliferative than **9a**, apoptosis induction, autophagy inhibition, G2-M phase arrest, degradation of cyclin B1 and Her2, prolonged survival of Eµ-Myc mice [64,65]
**10** (KBR1307)	MCF-7 breast carcinoma	More antiproliferative than **9a**, binds Hsp70 in absence of nucleotide [66]
**11** (AEAC)	C6 rat glioblastoma, B16 mouse melanoma	Increased doxorubicin activity in vitro and in vivo, tumor growth inhibition (71%) and prolonged survival in B16 mice [68]

**Table 3:** Grp78 inhibitors and their effects on cancer model(s).

Grp78 Inhibitor	Cancer Model(s)	Effects
**1** (VER-155008)	Osteosarcoma, MCF-7 and MDA-MB-231 breast cancer	Antiproliferative, apoptosis induction, suppression of tamoxifen-induced phosphor-GSK-3β [30,69]
**12**	HCT-116 colon carcinoma	More selective for Grp78 than **1**, no antiproliferative activity [70]
**13** (HA15)	Melanoma (BRAF-mutant), A549 NSCLC, KRAS-mutant cancer, adrenocortical carcinoma	ER stress, induction of apoptosis and autophagy, in vivo inhibition of A375 melanoma growth, suppression of KRAS and steroidogenesis, synergy with mitotane [71,72,73,74]
**14** (OSU-03012)	Carboplatin-resistant canine osteosarcoma (HMPOS-2.5R and HMPOS-10R), GBM5 and GBM12 glioblastoma	Antiproliferative, Bag2 suppression, formation of toxic autophagosomes [75,76,77]
**15** (YUM70)	KRAS-mutant cancer, pancreatic cancer (Mia-PaCa-2, PANC-1, BxPC-3)	Antiproliferative, ER stress, eIF2α phosphorylation, AT4 and CHOP activation, synergy with vorinostat and topotecan, in vivo MiaPaCa-2 tumor growth inhibition [73,78]
**16** (HM01), **17** (HM03)	HCT-116 colon carcinoma	Antiproliferative [79]
**18** (IKM5)	Breast cancer (MCF-7, MDA-MB-231, MDA-MB-468, BT474, 4T1)	Antiproliferative, suppression of MMP-2, Twist1 and vimentin, induction of TIMP-1 and Par-4, in vivo inhibition of breast tumor growth and lung metastasis formation [80]
**19** (FL5)	Renal cell carcinoma, HUVECs	Cell death, anti-angiogenic [81]
**20** (Hexachlorophene)	HCT-116 colon carcinoma	Cytotoxic, induction of apoptosis and autophagy, upregulated ATF4, XBP1s, and CHOP [82]
**21** (VH1019)	MCF-7 breast carcinoma	ATP-mimic, antiproliferative [83]
**22** (KP1339/BOLD-100)	Miscellaneous, HCT-116 colon carcinoma, REN pleural mesothelioma, Capan1 pancreatic carcinoma	Apoptosis induction and ER disruption in **22**-sensitive cells, G2 cell cycle arrest in **22**-resistant cells, binding to ribosomal proteins, ER stress, cytotoxic, ROS formation, induction of CHOP and XPB1, glycolysis upregulation, synergy with 2-deoxyglucose [85,86,87,88]

**Table 4:** Mortalin inhibitors and their anticancer effects.

Mortalin Inhibitor	Cancer Model(s)	Effects
**3b** (JG-98)	Multiple myeloma	Antiproliferative, 55S mitoribosome degradation [90]
**3c** (MKT-077)	Miscellaneous, ras-induced cancer, K562 leukemia	Antiproliferative, mitochondria accumulation, activation of p53 and p21, inhibition of mortalin-C9 [91,92,93,94,95,96,97]
**23**	Miscellaneous, HeLa cervix carcinoma	Antiproliferative, p53 and Bak activation [40]
**24a** (Mortaparib)	HeLa cervix and SKOV-3 ovarian carcinoma	Dual mortalin and PARP1 inhibitor, p53 activation, apoptosis induction, in vivo inhibition of SKOV-3 tumor growth and metastases [99]
**24b** (Mortaparib^Plus^)	HCT-116 and DLD-1 colon carcinoma	Inhibition of mortalin-p53 and PARP1, CARF-1 suppression, induction of apoptosis and p21 [100]
**24c** (Mortaparib^Mild^)	HCT-116 colon carcinoma	Inhibition of mortalin-p53 and PARP1 [102]
**25** (SHetA2)	Ovarian cancer	Inhibition of mortalin-p53, synergy with p53-reactivator PRIMA-1^Met^, caspase-activation, increased ROS formation, reduced ATP, in vivo inhibition of MESOV tumor growth, clinical phase 1 studies (advanced/recurrent cervical, endometrial and ovarian cancer) [15,103,104]

**Table 5:** Hsp40 modulators and their anticancer effects.

Hsp40 Modulator	Cancer Model(s)	Effects
**26a** (MAL3-101)	Merkel cell carcinoma, muscle invasive bladder cancer, RMS13 rhabdomyosarcoma	Antiproliferative, apoptosis induction, in vivo MCC growth inhibition, synergy with **1** and STA9090, UPR induction [110,111,112]
**28** (KNK437)	Squamous cell carcinoma (KB, SCC VII and SAS/mp53), immortalized Cos-1, MDA-MB-231 breast carcinoma, colon carcinoma (COLO 320DM, SW480, SW620, RKO, LOVO)	Suppression of Hsp70 and Hsp40, in vivo inhibition of SCC VII squamous cell carcinoma at 44 °C, apoptosis induction and colony formation inhibition at 42 °C, inhibition of heat-induced H3-Lys4 methylation, suppression of AKT and HIF-1α pathways, selective inhibition of DNAJA1, in vivo inhibition of DNAJA1-overexpressing SW480 and SW620 tumors, suppressed CDC45, upregulated ubiquitin, in vivo suppressed liver metastasis formation with 5-FU/L-OHP [113,114,115,116,117,118,119,120]
**29** (C86/SU086)	HeLa cervix carcinoma, 22Rv1 and C4-2 prostate cancer	Antiproliferative, pan-Hsp40/DNAJ inhibition, apoptosis induction, ROS formation, inhibition of thioredoxin reductase, degradation of FL-AR and ARv7, Hsp90 inhibition, in vivo 22Rv1 and C4-2 prostate tumor growth inhibition [121,122,123]
**30a** (PLIHZ), **30b** (PLTFBH)	HN31 pharyngeal squamous cell carcinoma	Antiproliferative, suppression of DNAJA1, mutant p53, Cdc42 and Rac [124]
**31** (Cabazitaxel)	LNCaP and PC-3 prostate cancer	Antiproliferative, suppression of Hsp40, HOP and AR [125]
**32** (Tipifarnib)	SF763 and U87 glioblastoma	Farnesyltransferase inhibition, reduction of farnesylated HDJ-2, radio-sensitizing, antiproliferative, p21 induction, G2/M arrest [131]
**33** (Atrovastatin)	Pancreatic carcinoma (PO3, SU 86.86, BXPC-3, Pan 10.05)	HMG-CoA reductase inhibition, suppression of DNAJA1 farnesylation, induction of apoptosis and p21, degradation of mutant p53 (blocked nuclear transport), inhibition of migration [132]
**34** (Marbostat-100)	MYC-overexpressing B-cell lymphoma	HDAC6 inhibition, apoptosis induction, MYC- degradation, tubulin hyperacetylation, relocation of DNAJA3 to acetyltubulin [133]
**35a**, **35b**	U2OS osteosarcoma	Activation of Hsp40 and p53, suppression of CRM1 [134]

## Data Availability

Not applicable.
